# Impact of Elderly Masticatory Performance on Nutritional Status: An Observational Study

**DOI:** 10.3390/medicina56030130

**Published:** 2020-03-16

**Authors:** Luca Aquilanti, Sonila Alia, Sofia Pugnaloni, Erminia Coccia, Marco Mascitti, Andrea Santarelli, Luisa Limongelli, Gianfranco Favia, Margherita Mancini, Arianna Vignini, Giorgio Rappelli

**Affiliations:** 1 Department of Clinical Specialistic and Dental Sciences, Polytechnic University of Marche, 60126 Ancona, Italy; l.aquilanti@pm.univpm.it (L.A.); s.alia@pm.univpm.it (S.A.); s.pugnaloni@pm.univpm.it (S.P.); dcermi@virgilio.it (E.C.); marcomascitti86@hotmail.it (M.M.); andrea.santarelli@staff.univpm.it (A.S.); g.rappelli@staff.univpm.it (G.R.); 2 Dentistry Clinic, National Institute of Health and Science of Ageing, IRCCS INRCA, 60126 Ancona, Italy; 3 Department of Interdisciplinary Medicine, University of Bari, 70124 Bari, Italy; luisanna.limongelli@gmail.com (L.L.); edottor@libero.it (G.F.)

**Keywords:** masticatory performance, BMI, waist circumference, bioimpedance

## Abstract

*Background and Objectives:* Masticatory limitations on the dietary habits of edentulous subjects restrict their access to adequate nutrition, exposing them to a greater risk of protein energy malnutrition. The aim of this study is to verify the existence of an association between Masticatory Performance (MP) and nutritional changes in the elderly. *Materials and Methods:* 76 participants were enrolled. MP testing was performed using the two-color chewing gum mixing test. The system used reveals the extent to which the two differently colored chewing gums mix, and allows discrimination between different MPs. The assessment of the participants’ nutritional statuses was carried out through a food interview. Anthropometric parameters were collected, and bioimpedance analysis was performed. *Results:* Mean MP was 0.448 ± 0.188. No statistically significant differences were detected between male and female subjects (*p* > 0.05). According to the Body Mass Index (BMI), obese patients had a lower MP than overweight and normal weight subjects (0.408 ± 0.225, 0.453 ± 0.169 and 0.486 ± 0.181, respectively). MP values were lower both in male and female subjects with a waist circumference above the threshold than those below it (0.455 ± 0.205 vs. 0.476 ± 0.110, respectively, in males and 0.447 ± 0.171 vs. 0.501 ± 0.138, respectively, in females). No relationship was noticed between MP and bioimpedance parameters (*p* > 0.05). *Conclusions:* A statistically significant relation was observed between MP and the number of missing teeth. A reduced MP could worsen nutritional parameters. A reduced MP did not seem to negatively affect bioimpedance parameters.

## 1. Introduction

According to recent studies [1,2], the percentage of elderly people will significantly increase over the next few decades. The increase in average life expectancy, attributable to the great socio-economic development of the last century, means an aging population and highlights the need to pay more attention to the health of these subjects.

Oral health is an important part of general health, affecting the quality of life [3], and it is therefore important to try to preserve it as much as possible, preventing and possibly treating all the problems most frequently encountered by the elderly that could lead to edentulism [4,5,6]. The latter is also the main cause of the reduction in masticatory performance (MP) and efficiency. According to previous studies, at least 20 teeth are necessary to maintain an adequate MP. The distribution and the number of teeth, as well as the quality and the type of oral rehabilitation, affect MP [7,8,9].

Nutritional status is an important health factor in elderly patients, and its assessment is crucial to prevent numerous acute and chronic diseases [10]. Oral disorders associated with reduced masticatory function negatively affect the nutritional status of the elderly [11]. The inability to chew and shred food properly tends to exclude some basic food from their diets, such as meat, fruit and vegetables, favoring the consumption of refined carbohydrates, fats or soft or overcooked food that risks losing most of its micronutrients [12].

The limitations on the dietary habits of edentulous subjects do not allow them to have adequate nutrition, exposing them to a greater risk of protein energy malnutrition (PEM) than their peers with an adequate number of natural teeth [13].

As stated by Yoshida et al. [14], tooth loss leads to a change in diet and could, therefore, be linked to eating disorders such as obesity, undernutrition and malnutrition. Nevertheless, it is not clear if changes in eating habits and the intake of certain food may determine the onset of conditions that could lead to edentulism and to a further reduction of the masticatory function.

According to Tada and Miura [15], an association between reduced chewing performance and obesity was shown. This could be linked to the fact that reduced masticatory function leads the subject to consume a greater amount of soft food, including food rich in fats or refined carbohydrates, and reduce the intake of fruits, vegetables and meat. However, a high consumption of sweet food in elderly subjects may be due to many other causes, such as the reduction of gustatory and olfactory perception, as well as economic and psychological factors [12,16,17].

The aim of this study is to verify the existence of an association between the reduction of MP in the elderly with different degrees of edentulism and the presence of nutritional changes.

## 2. Materials and Methods

Participants were enrolled among patients undergoing medical outpatient treatment at the Dental Clinic of the Marche Polytechnic University of Ancona, during the period from April 2017 to December 2017. 

Inclusion criteria were: (a) ≥ 65 years old; (b) self-sufficient subjects.

Exclusion criteria were: (a) diagnosis of neurodegenerative diseases (e.g., Alzheimer, Parkinson, dementia); (b) history of strokes; (c) diagnosis of diseases and disorders affecting the muscular system; (d) orofacial pain; (e) diagnosis of xerostomia and/or Sjogren syndrome; (f) clinical conditions that require a specific diet (e.g., type 2 diabetes mellitus, coeliac disease).

The study was performed in accordance with the principles of the Declaration of Helsinki as revised in 2013, and it was approved by the Local Institutional Review Board (Project identification code: OD2017 01, Date of Approval: 03-16-2017, Dental Clinic Local Institutional Review Board, Ancona, Italy). Written, informed consent was obtained from all participants.

All the patients underwent dental examination. For all participants, individual sociodemographic data, as well as general and oral health data, were recorded. 

**Figure 1:**
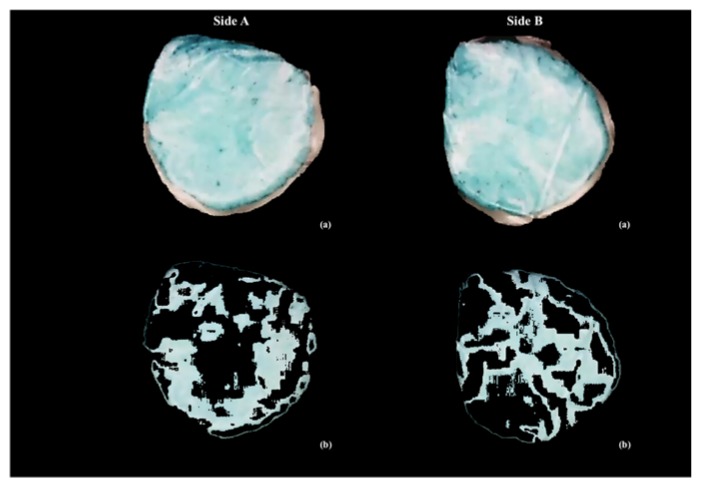
Digital analysis of a sample: (**a**) flattened bolus side A and side B; (**b**) mixed bolus side A and side B.

A masticatory test was carried out, using the two-color mixing test as described by Schimmel et al. [18,19]. Briefly, the test involves the use of two colored chewing gums (Hue-check Gum®, Orophys GmbH, Muri b. Bern, Switzerland). Each sample was chewed for 20 chewing cycles, as this number of strokes allows the assessment of MP. Boluses were collected, inserted between two sheets of transparent plastic and brought to a standard thickness of 1 mm. Standardized photos were taken from both sides of each bolus, and all the obtained images were processed by computer, analyzing the measure of the area of pixels of different colors using the K-means clustering method [20]. At the end of the analysis, the software revealed the extent to which the chewing gums mixed, and allowed discrimination between the different MPs of the subjects (Figure 1). 

Participants underwent both a qualitative and a quantitative food interview to assess their nutritional status. Participants communicated the type of food they generally consume in each meal and the relative quantities in grams. The obtained food data were entered into the appropriate software (WinFood, Medimatica, Martinsicuro, Italy) through which the percentages of carbohydrates, lipids and proteins that the patient consumed were shown, based on the Italian food composition tables [21,22]. 

Weight (in kg), height (in cm) and waist circumference (WC) (in cm) were measured. Body weight was measured, without shoes and wearing minimal clothes, with a scale to the nearest 0.01 kg. Height was measured to the nearest 0.1 cm with a stadiometer (Seca, Hamburg, Germany) at enrolment, according to standardized procedures described elsewhere [23]. WC was measured with a metric band along a horizontal plane parallel to the floor. It was considered as the minimum circumference between the rig cage and the navel, with the subject standing with relaxed abdominal muscles.

Then, a bioimpedance analysis was performed with a DF50 Body Composition Analyzer (ImpediMed, Brisbane, Australia). The bioimpedance measurement represents the resistance of the body to the passage of a low intensity electric current (800 μA) at high frequency (50 kHz) and is performed by placing a pair of electrodes, connected to the measuring instrument, on the back of the hand and another two on the back of the foot. Impedance (Z), Resistance (R), Phase Angle (Ph) and Reactance (Xc) were evaluated. 

Statistical analysis was performed using the statistical software R (R version 3.5.3).

## 3. Results

During the period April 2017 to December 2017, 217 subjects attended the Dental Clinic. Only 76 individuals met the inclusion criteria and were enrolled in this study. They were 42 men and 34 women aged over 65 years (mean age: 75.8 ± 5.6). The patients’ medical histories revealed the presence of some systemic diseases, such as: hypertension (55 subjects), osteoarthrosis (18 subjects), heart failure (13 subjects), chronic obstructive pulmonary disease (9 subjects), and Hashimoto’s disease (1 subject). No association was detected between MP and those pathologies (*p* > 0.05). 

Dental examination revealed that 68.5% of the participants had more than 20 teeth, with a mean of 21.8 ± 5.7 teeth, with a mean of missing teeth of 6.16 ± 5.66 and with a mean number of occluding pairs of 10.5 ± 2.9. Of the participants, 48.7% (*n* = 37) declared that they had no drinking habits, 38.2% (*n* = 29) declared that they generally consumed less than or equal to 2 daily units of alcoholic drink equivalent and 13.1% (*n* = 10) more than 2. Further, 43.4% (*n* = 33) of the participants were nonsmokers, 10.5% (*n* = 8) were former smokers, 23.7% (*n* = 18) generally smoked less than 10 cigarettes or equivalent per day and 22.4% (*n* = 17) more than 10. A Full Mouth Plaque Score (FMPS) was assessed for each participant, considering six surfaces per tooth and having set 20% as the cut-off. Results showed that 31 subjects had an FMPS lower than 20% and 45 had an FMPS equal to or higher than 20%. The epidemiological and clinical data are reported in Table 1.

**Figure 2:**
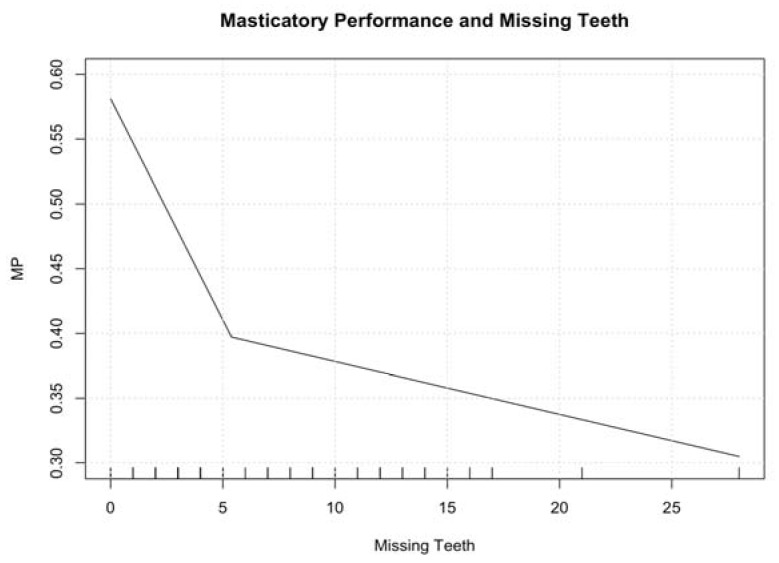
Masticatory performance by number of missing teeth.

**Figure 3:**
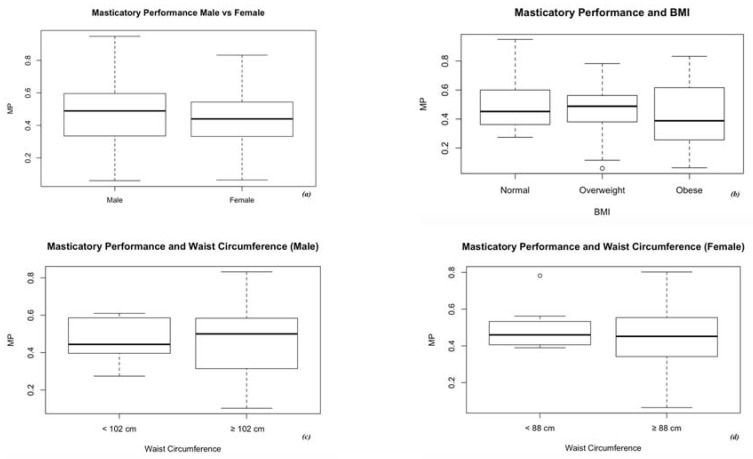
Participants’ masticatory performance in relation to: (**a**) gender, (**b**) BMI, (**c**) waist circumference in males and (**d**) waist circumference in females.

The MP of the 76 participants in relation to the number of lost teeth was evaluated. Results are shown in Table 2 and Figure 2. The MP was 0.45 ± 0.19, showing no statistically significant differences between males and females (0.46 ± 0.20 vs. 0.43 ± 0.18, respectively), as shown in Figure 3.

**Figure 4:**
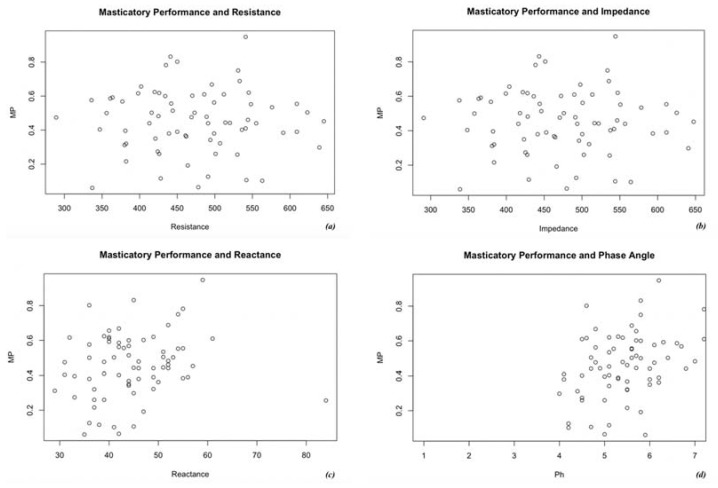
Participants’ masticatory performance in relation to bioimpedance parameters such as: (**a**) resistance, (**b**) impedance, (**c**) reactance and (**d**) phase angle.

The participants were divided into three groups according to BMI. Of the subjects, 22 were within the normal weight range (18.5 ≤ BMI < 25), 39 were overweight (25 ≤ BMI < 30) and 24 obese (BMI ≥ 30). For each group, MP was evaluated (Figure 4). Even though obese patients had a lower MP (0.41 ± 0.23) than both overweight (0.45 ± 0.17) and normal weight (0.49 ± 0.18) patients, a statistically significant difference was not observed among the groups. 

As WC has two different cut-offs according to the sex of the patient [24], the participants were divided into groups on the basis of WC. The relationship between MP and WC in male and female subjects was evaluated, and the results are shown in Figure 3. In the male population, subjects with a WC above the threshold had an MP lower than those under it (0.46 ± 0.21 vs. 0.48 ± 0.11). Likewise, in the female population those who had a WC above the threshold had a lower MP than those under it (0.45 ± 0.17 vs. 0.50 ± 0.14). Also in this case, the data did not reach a statistically significant difference. 

The evaluation was repeated, taking as reference values the parameters of Resistence (R), Impedence (Z), Phase Angle (Ph) and Reactance (Xc). Results are shown in Figure 4. According to statistical analysis, no relationship was noticed between MP and any of the studied parameters (*p* > 0.05). 

## 4. Discussion

The study was performed on a sample of 76 patients, who underwent a dental examination during which a masticatory test was performed using the two-color mixing test, and who participated in a nutritional interview.

Comparing the data related to the chewing performance test, it was found that, in accordance with the literature [7,8,9], MP in subjects with less than 20 teeth is lower than in those with more than 20. In particular, Figure 2 shows how dramatically the line representing MP decreased from 0 to 5 missing teeth. Then, the line decreased moderately. 

Sheiham et al. [17] investigated the possible relation among oral conditions, the intake of selected nutrients and blood-derived values of key nutrients in adults aged over 65 years old. It was demonstrated that a lower nutrient intake was associated with a higher number of missing teeth. In particular, being edentulous was statistically related to the intake of several key nutrients. Food selection could prevent edentulous subjects, and in general subjects with less than 20 teeth, from having adequate nutritional status. In the sample, even if subjects defined as obese had a lower MP than the ones defined as overweight and normal weight, a statistically significant difference among the groups was not found. Moreover, in the sample there were no underweight subjects (BMI <18.5). So, it was not possible to evaluate the influence of MP on undernutrition. Also, comparing MP and WC in male and female populations, no statistically significant differences were detected between subjects within the physiologic range and the ones above it, even if MP was lower in the group of participants with WC above the threshold. A possible explanation for these results lies in the fact that the analyzed sample consisted of independent and self-sufficient subjects.

MP was also evaluated in relation to bioimpedance. Bioimpedance is a non-invasive method that analyzes tissue properties and provides reliable information about body composition by the transmission of a series of alternating electric currents through the body. In a previous study it was proven that bioimpedance parameters are more appropriate for nutritional status assessment than BMI [25]. No association among MP and Z, R, Ph or Xc was detected in this study. Nevertheless, it is important to consider that the reduction of lean mass is frequent in elderly patients and is linked to PEM [26]. The latter may also be more pronounced in subjects with high masticatory deficits. The difficulty in chewing meat may lead to a lower consumption of proteins that is not always offset by the intake of other protein bearing foods that are more easily chewed [12,17]. As reported in previous studies, cases of undernutrition and malnutrition are more frequent in institutionalized or hospitalized subjects. Taste and swallowing difficulties, bad oral conditions, drug consumption, constipation, neurodegenerative diseases and reduced daily life activities are some of the factors that are considered to be strongly related to malnutrition or to the risk of it [13,27,28,29,30]. 

Another aspect that could affect MP is sarcopenia, defined as a generalized and progressive skeletal muscle disorder that is linked to an increased likelihood of adverse outcomes such as falls, fractures, physical disability and mortality [31]. It not only has serious consequences for general health but also affects the oral component, thus reducing the strength of the muscles involved in chewing and swallowing. Musculoskeletal mass loss in subjects with sarcopenia also affects oral musculature, with an associated nutritional deterioration and a worsening of the sarcopenic pathology itself [32,33]. Sarcopenia is generally associated with ageing, worsens general health status and increases the risk of hospitalization. It would, therefore, be interesting to extend the sample to institutionalized or hospitalized elderly patients in the future.

## 5. Conclusions

The sample consisted exclusively of independent and self-sufficient patients without serious impairment of the general state of health and, apart from a few cases, most of them presented a good oral condition. An association between reduced MP and a worsening of nutritional parameters was not revealed in the results of this study. Furthermore, MP seemed not to negatively affect bioimpedance parameters such as R, Z, Ph and Xc.

Not surprisingly, a statistically significant relation was observed among MP, the number of missing teeth and the number of occluding pairs. Nevertheless, due to the small sample size and the possible adaptive capacity, the results are not conclusive and further studies are needed. 

## Figures and Tables

**Table 1:** Epidemiological and clinical data.

	Male	Female		
**Sex**	*42*	*34*		
**Age**	Mean	SD		
***-Total***	*75.8*	*5.6*		
***-Male***	*76.0*	*6.0*		
***-Female***	*75.8*	*6.0*		
**N° Teeth**	Mean	SD		
	*21.8*	*5.7*		
**N° Occluding Pairs**	Mean	SD		
	*10.5*	*2.9*		
**Alcohol ^a^**	No	≤ 2	>2	
	*37*	*29*	*10*	
**Smoking Habits**	No	Former ^b^	<10	≥10
	*33*	*8*	*18*	*17*
**Oral Hygiene ^c^**	<20%	≥20%		
	*31*	*45*		

^a^ Daily Units of alcoholic drink equivalent; ^b^ ≥ 5 years; ^c^ Full Mouth Plaque Score.

**Table 2:** Results of Masticatory Performance by Sex, BMI and Waist Circumference. Data are expressed as mean and standard deviation (SD).

Sex	MP ^a^	SD	*p*-Value
*-Male*	*0.46*	*0.20*	
*-Female*	*0.43*	*0.18*	
			*>0.05*
**BMI ^b^**	MP	SD	
*-Normal*	*0.49*	*0.18*	
*-Overweight*	*0.45*	*0.17*	
*-Obese*	*0.41*	*0.23*	
			*>0.05*
**WC ^c^ Male**	MP	SD	
*-<102 cm*	*0.48*	*0.11*	
*- ≥ 102 cm*	*0.46*	*0.21*	
			*>0.05*
**WC Female**	MP	SD	
*- < 88 cm*	*0.50*	*0.14*	
*- ≥ 88 cm*	*0.45*	*0.17*	
			*>0.05*

^a^ Masticatory Performance; ^b^ Body Mass Index; ^c^ Waist Circumference.
